# Characterization of Mechanical and Biological Properties of 3-D Scaffolds Reinforced with Zinc Oxide for Bone Tissue Engineering

**DOI:** 10.1371/journal.pone.0087755

**Published:** 2014-01-31

**Authors:** Pei Feng, Pingpin Wei, Cijun Shuai, Shuping Peng

**Affiliations:** 1 State Key Laboratory of High Performance Complex Manufacturing, Central South University, Changsha, Hunan Province, P. R. China; 2 Department of Regenerative Medicine & Cell Biology, Medical University of South Carolina, Charleston, South Carolina, United States of America; 3 Cancer Research Institute, Central South University, Changsha, Hunan Province, P. R. China; 4 Department of Obstetrics, Gynecology and Reproductive Sciences, Yale University School of Medicine, New Haven, Connecticut, United States of America; Politecnico di Milano, Italy

## Abstract

A scaffold for bone tissue engineering should have highly interconnected porous structure, appropriate mechanical and biological properties. In this work, we fabricated well-interconnected porous β-tricalcium phosphate (β-TCP) scaffolds via selective laser sintering (SLS). We found that the mechanical and biological properties of the scaffolds were improved by doping of zinc oxide (ZnO). Our data showed that the fracture toughness increased from 1.09 to 1.40 MPam^1/2^, and the compressive strength increased from 3.01 to 17.89 MPa when the content of ZnO increased from 0 to 2.5 wt%. It is hypothesized that the increase of ZnO would lead to a reduction in grain size and an increase in density of the strut. However, the fracture toughness and compressive strength decreased with further increasing of ZnO content, which may be due to the sharp increase in grain size. The biocompatibility of the scaffolds was investigated by analyzing the adhesion and the morphology of human osteoblast-like MG-63 cells cultured on the surfaces of the scaffolds. The scaffolds exhibited better and better ability to support cell attachment and proliferation when the content of ZnO increased from 0 to 2.5 wt%. Moreover, a bone like apatite layer formed on the surfaces of the scaffolds after incubation in simulated body fluid (SBF), indicating an ability of osteoinduction and osteoconduction. In summary, interconnected porous β-TCP scaffolds doped with ZnO were successfully fabricated and revealed good mechanical and biological properties, which may be used for bone repair and replacement potentially.

## Introduction

Scaffold plays an important role in cells attachment, proliferation and guidance of new bone tissue formation in bone tissue engineering [1]–[2]. Highly interconnected porous structure and customized shape for specific bone defects are essential for ideal bone scaffold. Though porous scaffold with sufficient void volumes and surface area can be obtained by some conventional methods including freeze drying, gas foaming, solvent casting, and so on [3]–[5], it is difficult to realize the precise control of the pore size, pore geometry and spatial distribution or the ability to construction of internal interconnected pore network [6]–[8]. SLS is an additive manufacturing technique in which a high power laser was used to sinter powder material selectively, and the latter become three-dimensional (3D) objects. The sintering process is guided by the computer aided design (CAD) model and computer control [9]–[10]. By this technique, the bone scaffolds with well-controlled internal architectures and customized external shapes can be well realized [11].

In addition to porous structure requirements, the scaffold should also have good biocompatibility, biodegradability and adequate mechanical properties [12]. β-TCP [β-Ca_3_ (PO_4_)_2_] is one of the most attractive bioceramics to produce the bone scaffold for tissue engineering because of the favorable biological compatibility, osteoconductivity and resorbability [13]. However, the low mechanical strength and toughness have restricted the application only in low- or non-load-bearing bone replacement [14]. Further, the degradation rate of pure β-TCP is too fast and uncontrolled which leads to mismatch between the degradation of scaffold and the formation of new bone [15]. Thus, it is necessary to improve mechanical and biological properties for the porous β-TCP scaffold to support bone regeneration.

It is reported that the mechanical properties and the degradation rate of β-TCP can be reinforced and modified by oxide-based dopant (such as ZnO, MgO, SiO_2_) without altering its inherent biocompatibility, respectively [16]–[17]. Zinc (Zn) was an essential trace element with a stimulatory role in bone formation and their mineralization [18]. In vitro studies showed that Zn had a direct specific proliferative role for osteoblastic cells [19] and a selective inhibitory role for osteoclastic bone resorption [20]. Jin et al. [21] produced hydroxyapatite/TCP biphasic bioceramics reinforced with ZnO-whiskers via conventional sintering and found that the addition of ZnO improved the hardness and fracture toughness. Bandyopadhyay et al. [22] fabricated TCP samples with different content of ZnO by uniaxial pressing followed by muffle furnace sintering and found that ZnO addition increased densification and hardness of ceramics. Bhatt et al. [23] conducted experiment to study the biodegradation rate of four different oxide-based β-TCP bioceramics in dynamic SBF and found that the ZnO addition could tailor the degradation rate of β-TCP. Bandyopadhyay et al. [24] had made TCP-ZnO compacts and the compacts showed an increase in densification and excellent biocompatibility with osteoblastic precursor cell line 1 cells compared with pure TCP. These investigations showed that oxide-based dopant such as ZnO could improve the mechanical properties and delay the degradation rate of β-TCP.

In this study, 3D porous bone scaffolds with controlled interconnected porous structure based on β-TCP powder with or without ZnO doping was fabricated via SLS technique. The degradation and bioactivity behavior of these porous scaffolds were studied with respect to weight loss and apatite layer formation. The in vitro biocompatibility was evaluated by the proliferation of MG-63 cells cultured on the surface of the porous scaffold. The surface morphologies were characterized using scanning electron microscope (SEM). The phase composition of the raw β-TCP powder and prepared scaffolds with different ZnO content was characterized by X-ray diffraction (XRD). The chemical composition of the scaffolds before and after SBF was analyzed using Fourier transform infrared spectrometer (FT-IR). The hardness and fracture toughness were determined by Vickers microindentor. Compressive tests were performed using an electronic universal test machine. Elemental composition of the deposits on the scaffold surface after SBF was analyzed by energy dispersive X-ray spectroscopy (EDX) spot analysis.

## Materials and Methods

### Materials

Raw β-TCP powder with the average particle size of 0.1∼0.3 µm was acquired from Kunshan Chinese Technology New Materials Co., Ltd. High-purity ZnO powder (99.9% purity) with the average particle size of 0.1∼0.2 µm was purchased from Guangdong Xilong Chemical Co., Ltd for this research. ZnO was added to β-TCP powder in proportions of 0.5, 1.5, 2.5 and 3.5 wt%, followed by mechanical mixing for 30 min. Pure β-TCP powder (i.e., 0 wt% ZnO) was also prepared and used as the control sample.

### Fabrication

**Figure 1:**
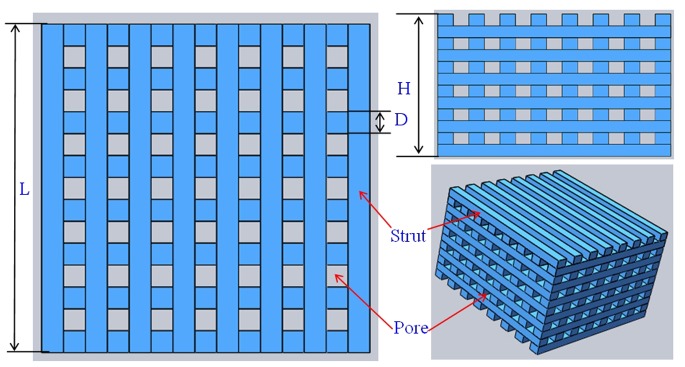
Scaffold model designed using SolidWorks software: D = 1.2 mm; H = 11 mm; L = 18 mm.

The scaffolds were prepared using a homemade selective laser sintering system [25]. The fabrication process began by spreading a thin layer of powder evenly onto the flat surface. The thin layer of powder could be formed by roll compaction. Then, the laser was guided to sinter the particles selectively in a powder bed layer-by-layer to fabricate the 3D scaffold based on the instructions of CAD program. A tetragonal porous scaffold model (L×W×H = 18×18×11 mm^3^, L is the length of scaffold, W is the width of scaffold, H is the height of scaffold) with 3D orthogonal periodic porous architecture was designed using SolidWorks software (Fig. 1). The tetragonal porous scaffold model was composed of eight struts per set, with each strut of 1.2 mm in width, 0.92 mm in thickness and 2.4 mm in distance between the centerlines of the struts. The laser used in this research was a 100 W CO_2_ laser (model Firestar® t-Series, Synrad Co., USA). The sintering process was performed at the power of 12 W, the scanning speed of 100 mm/min, the spot diameter of 1.2 mm and the layer thickness of 0.1–0.2 mm. After the SLS processing was completed, the scaffolds were allowed to cool to room temperature before being removed from the powder bed. Excess powder was brushed off from the exterior while the internal architecture was cleaned using compressed air.

### Characterizations

The microstructures of the prepared scaffolds were examined by SEM (JEOL JSM-5600LV, JEOL Ltd., Japan). To study the effect of ZnO on grain size, the prepared samples (18×1.2×0.92 mm^3^) were etched with 5% hydrofluoric acid (HF) for 5 min and then coated with a very thin layer of gold. The average grain sizes were determined from SEM images with the linear intercept method using Eq. (1) [26]:
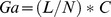
(1)Where 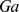 is the average grain size (µm), *L* is the total test line length (cm), *N* is the total number of intersections with grain boundaries along test line *L*, and *C* is the conversion factor (µm/cm) of the SEM picture on which the test lines were drawn as obtained from the scale bar. The phase composition was identified by XRD (D8-ADVANCE, German) with CuKα radiation (λ = 1.54056 Å) at 40 kV and 40 mA. The XRD patterns were recorded in the 2θ range of 10 to 40 degrees with a scanning rate of 8 degrees/min. Infrared spectra for the raw powder and the scaffolds were obtained using FT-IR (Thermo Scientific Nicolet™ 6700, Thermo Electron Corp., USA). For this purpose, the scaffolds were first grinded into powders, and then the powders were mixed with KBr in the proportion of 1/150 (by weight) for 15 min and pressed into a pellet using a cold press. Each infrared spectrum was obtained over the range from 500 to 4000 cm^−1^ with a resolution of 4.0 cm^−1^.

The density of the struts (18×1.2×0.92 mm^3^) was measured using the Archimedes method. The densification was calculated as the density divided by the theoretical density of the struts. The hardness and the fracture toughness were tested by Vickers Microindentor (Digital Micro Hardness Tester, HXD-1000TM/LCD, Shanghai Taiming Optical Instrument Co. Ltd) both with a load of 300 gf and a dwelling time of 15 s. The surface of strut samples (1×1.2×0.92 mm^3^) was mounted in epoxy vertically, polished and subjected to indentation on the surfaces. The Vickers hardness (*HV*) and fracture toughness (*K_IC_*) were calculated from the following Eq. (2) and (3), respectively:
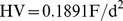
(2)

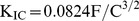
(3)Where *HV* is the Vickers hardness, *K_IC_* is the fracture toughness, *F* is the applied load, *d* is the diagonal of the indentation, and *C* the radial crack length measured from the center point of the indentation impression. Density and Vickers indentation tests were analyzed with six struts for each group. Compressive tests of the prepared scaffolds (18×18×11 mm^3^) were performed at a crosshead speed of 0.5 mm/min using a microcomputer control electronic universal test machine (WD-D1, Shanghai zhuoji instruments Co. Ltd) to evaluate the compressive strength and stiffness. All compression tests were performed in a direction perpendicular to the circular platens. The stress versus strain responses of the scaffolds was recorded to calculate the scaffold stiffness during the compressive strength tests. Six samples for each group were used to obtain the average value along with their standard deviation.

### Cell Culture

To evaluate the cell biocompatibility, human osteoblast-like MG-63 cells (American Type Culture Collection, Rockville, MD) were seeded on the prepared scaffolds (18×18×11 mm^3^). MG-63 cells were seeded in culture flasks in Dulbecco’s modified Eagle’s medium (DMEM; Cell- gro by Mediatech, Inc., Manassas, VA) containing 10% fetal bovine serum (Gibco, Carlsbad, CA) and 1% penicillin/streptomycin (Gibco) at 37°C, 5% CO_2_ and 100% humidity. The medium was changed every 2 days to wash off all non-adherent cells. The morphology of primary MG-63 cells was observed using a light microscope (Olympus, IX51, Japan). Prior to cell seeding, scaffolds were pre-wetted by immersing in 70% ethanol for 2 hours and exchanged by excess of phosphate buffered saline (PBS) solution. After the removal of PBS, 4×10^5^ cells were seeded onto each scaffold and cultured for 5 days in DMEM. Then the scaffolds were rinsed in PBS for three times for each sample, and then fixed with a 2.5% glutaraldehyde solution for 30 min. The scaffolds were then dehydrated by a series of graded ethanol solutions before critical point dried. After dried, the scaffolds were coated with a very thin layer of gold, and the cell adhesion on the scaffolds was assessed using SEM (TESCAN, Mira 3 FEG, Tescan Co.). The MG-63 cells cultured on the scaffold and dish were digested and seeded on cover slips in six-well dishes. After incubation for 24 h under standard culture condition, the cells were washed twice with D-PBS solution. Add sufficient amount of Calcein AM/EthD-III (Invitrogen Ltd., UK) standard staining solution to cover cell monolayer. The cells were incubated for 30–45 minutes at room temperature. Calcein AM/EthD-III solution was aspirated out after incubation. Ten micro liter of the fresh Calcein AM/EthD-III solution or PBS was added to a clean microscope slide, and the cover slips seeded with the cells was reverted and mounted on the prepared slides. Fingernail polish was used to seal the cover slips and to prevent evaporation. The labeled cells were observed under the fluorescence microscope (Olympus BX51, Japan).

### SBF Test

In order to evaluate the in vitro degradation behavior and bioactivity, all scaffold samples (15×15×7 mm^3^) with different ZnO content were immersed in a dynamic SBF at pH 7.4. SBF was an a cellular solution with an ionic composition (in units of mM, 142.0 Na^+^, 5.0 K^+^, 1.5 Mg^2+^, 2.5 Ca^2+^, 147.8 Cl^−^, 4.2 HCO_3_^−^, 1.0 HPO_4_^2−^ and 0.5 SO_4_^2−^) almost equal to that of human plasma and buffered at a similar pH. Six scaffolds for each group were soaked in the SBF solution under dynamic condition in a thermostat at 37°C for 7, 14, 21, and 28 days, respectively. The SBF solutions were refreshed every 3 days over the course. At a predetermined time interval, the scaffolds were taken out from the SBF solution, washed with distilled water and dried at 65°C in an incubator for 2 days. Once dried, the weight of the scaffolds was carefully recorded and compared with their initial weight before immersion. The biodegradation of the scaffolds was calculated by measuring their weight loss as a function of time. The weight loss (*WL*) of sample was calculated according to Eq. (4) [27]:
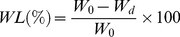
(4)Where *W_0_* is the weight before immersion in SBF and *W_d_* is the weight of the samples dried at 65°C for 2 days after removed from the SBF. Surface microstructures of these scaffolds were observed using SEM analysis to determine mineralization on the top layer. Elemental composition analysis was conducted by coupled EDX with SEM. The scaffolds surface after soaking was examined by FT-IR to confirm the formation of apatite layer.

### Statistical Analysis

All experimental data were expressed as means ± standard deviation and analyzed using SPSS software. Statistical analysis was performed using Student’s *t*-test and differences were considered significant for *P<0.05 and very significant when **P<0.01, respectively.

## Results and Discussion

### Microstructure

**Figure 2:**
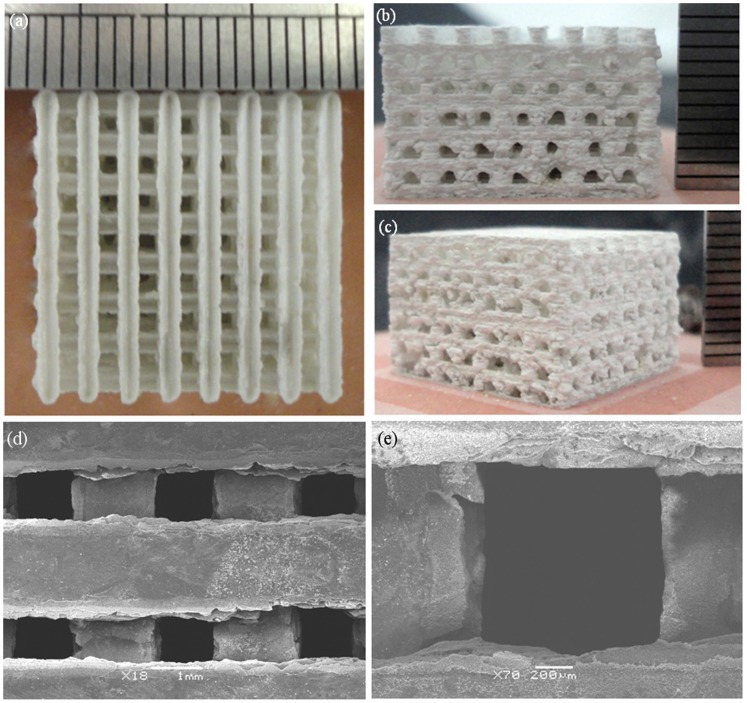
Optical micrographs of 3D interconnected porous scaffold prepared by SLS and SEM images of the porous structure (a) top view and (b–c) side view (d) the porous structure and (e) a single pore structure.

The prepared scaffold was approximately 18×18×11 mm^3^ in size (Figs. 2a–c). The porosity of the scaffolds was 56.8%. The scaffolds had well-ordered and uniform pore channel structures (Fig. 2d). There was good bonding between the adjacent layers of the scaffold. The width of the struts was 1.2 mm. The pore size of the scaffold is around 1.2 mm (Fig. 2e). The regular spatial arrangement pore channels were expected to provide osteoinductivity and guided bone tissue to grow well into the pores.

**Figure 3:**
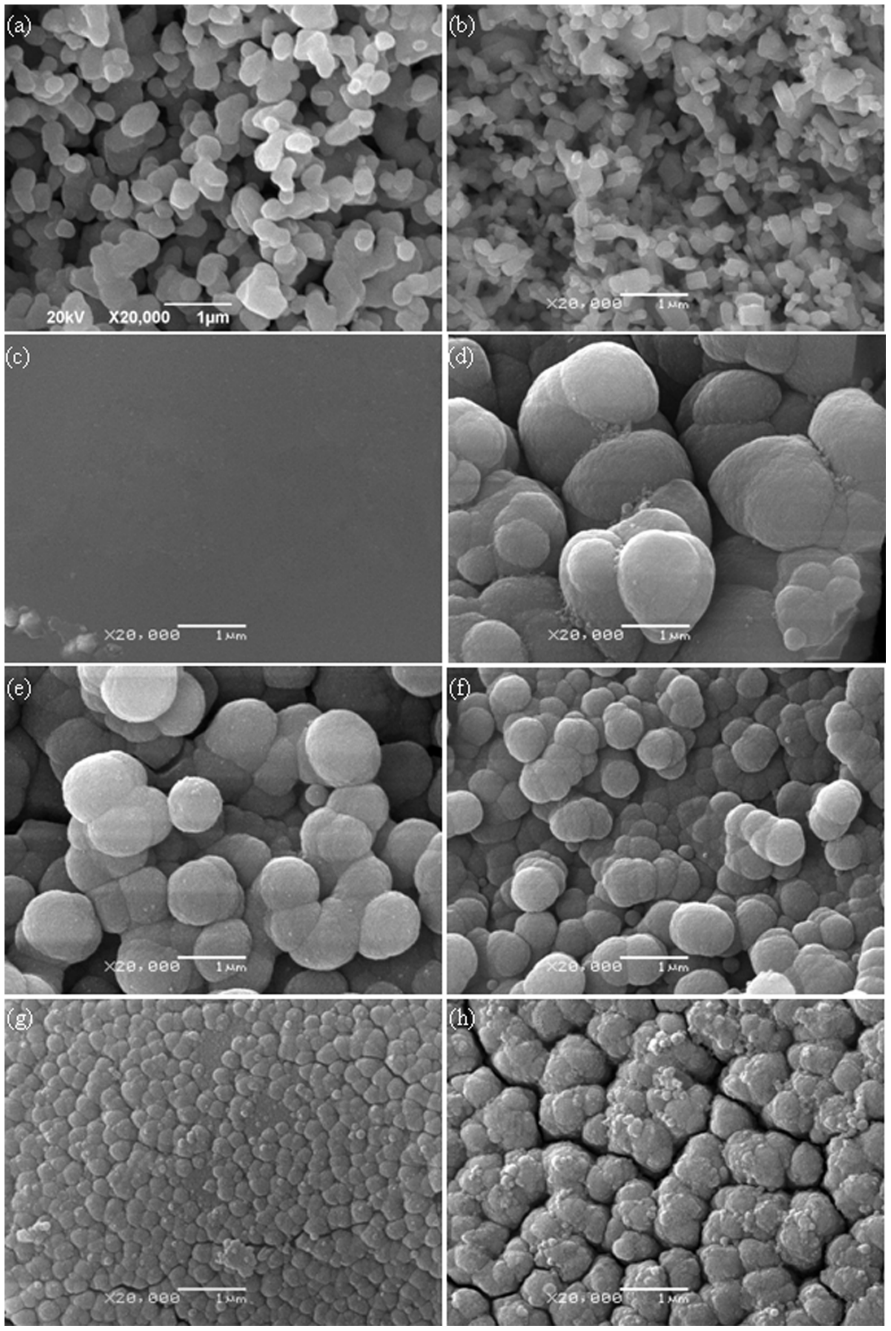
Micrographs of (a) raw β-TCP powder; (b) raw ZnO powder; (c) unetched surface of scaffold; etched surface of scaffold: (d) pure TCP; (e) 0.5 wt% ZnO; (f) 1.5 wt% ZnO; (g) 2.5 wt% ZnO and (h) 3.5 wt% ZnO.

The raw powder, non-etched and etched surface of the scaffold with different ZnO content were shown in Fig. 3. It could be observed that the raw β-TCP powder (Fig. 3a) and ZnO powder (Fig. 3b) were non-uniform and micro-particle loose aggregation. The average grain size of the raw β-TCP and ZnO powder was 0.1–0.3 µm and 0.1–0.2 µm, respectively. The non-etched surface revealed a smooth and highly dense structure (Fig. 3c). The etched scaffolds surface was showed in Figs. 3d–h. Micrographs of the samples showed clear demarcation in the grain boundaries. So their grain size could be easily calculated. The average grain size of the β-TCP scaffold without ZnO doping was calculated to be 1.54 µm. For the addition of 0.5 wt% ZnO, the average grain size dropped to 1.01 µm. At 1.5 wt% and 2.5 wt%, the average grain size decreased from 0.72 µm to 0.29 µm, respectively. The average grain size decreased due to ZnO was a refractory metal oxide (melting point: 1,975°C), which had an inhibitory effect on grain grow during the sintering process. While a sudden increase in grain size was observed when ZnO content is 3.5 wt%, the average grain size was 0.83 µm. β-TCP has a hexagonal crystal structure and belongs to R3CH space group. The unit cell dimensions are: a = b = 10.4183(5) Å, c = 37.3464(23) Å, and α = β = 90°, γ = 120° [28]. The Ca^2+^ in β-TCP can be substituted by Zn^2+^ during the sintering process. The presence of Zn^2+^ in β-TCP could decrease both the *a*-axis and *c*-axis lattice when the content of ZnO increased from 0 to 2.5 wt%. The *a*-axis lattice further decreased, while the *c*-axis lattice increased when the content of ZnO was over 2.5 wt%. The sharp increase in grain size was due to the increase of the c-axis lattice. It was reported that the c-axis lattice would increase when the Zn^2+^ content in the β-TCP was higher than certain content [13].

### Phase Composition

**Figure 4:**
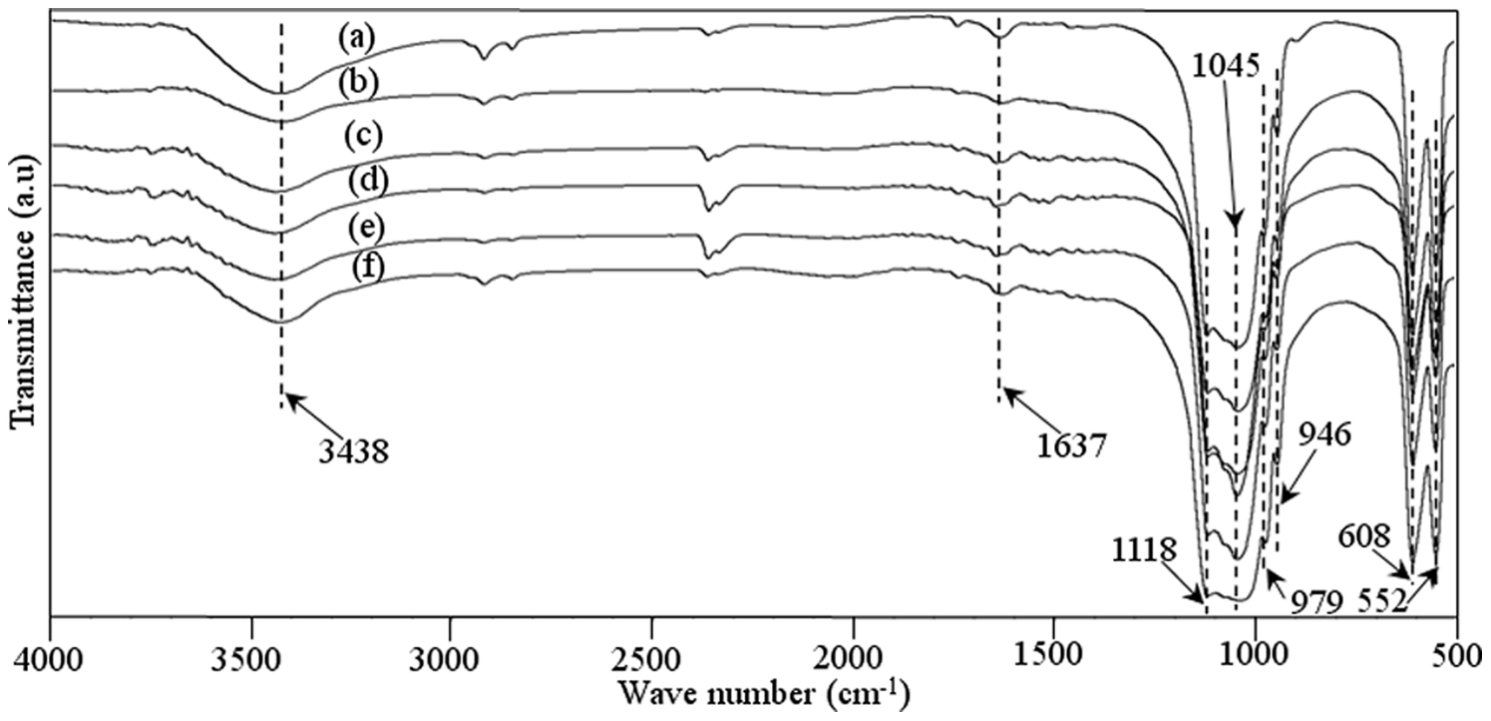
FT-IR spectra of the raw β-TCP powder (a) and β-TCP scaffolds with 0 wt% ZnO (b), 0.5 wt% ZnO (c), 1.5 wt% ZnO (d), 2.5 wt% ZnO (e), and 3.5 wt% ZnO (f).

The FT-IR spectrum of raw β-TCP powder and the prepared scaffolds was presented in Fig. 4. The FT-IR pattern of raw pure β-TCP powder was shown in Fig. 4a. The characteristic absorption bands at 3438 and 1637 cm^−1^ were attributed to adsorbed water. The bands at 900–1200 cm^−1^ were the stretching vibration mode of the PO_4_^3−^ group. The sharp peaks at 552 and 608 cm^−1^ represented the bend vibration mode of PO_4_^3−^ in β-TCP. The FT-IR pattern of the scaffolds with 0–3.5 wt% ZnO doped was shown in Figs. 4b–f. The characteristic peaks for β-TCP at wave numbers of 1118, 1045, 609, and 552 cm^−1^ were present. It was observed that FT-IR spectrum of raw β-TCP powder and the scaffolds showed typical spectrum for TCP material. There was no presence of new functional group in the prepared scaffolds, indicating that the doping of ZnO did not change the chemical composition of TCP after sintering.

**Figure 5:**
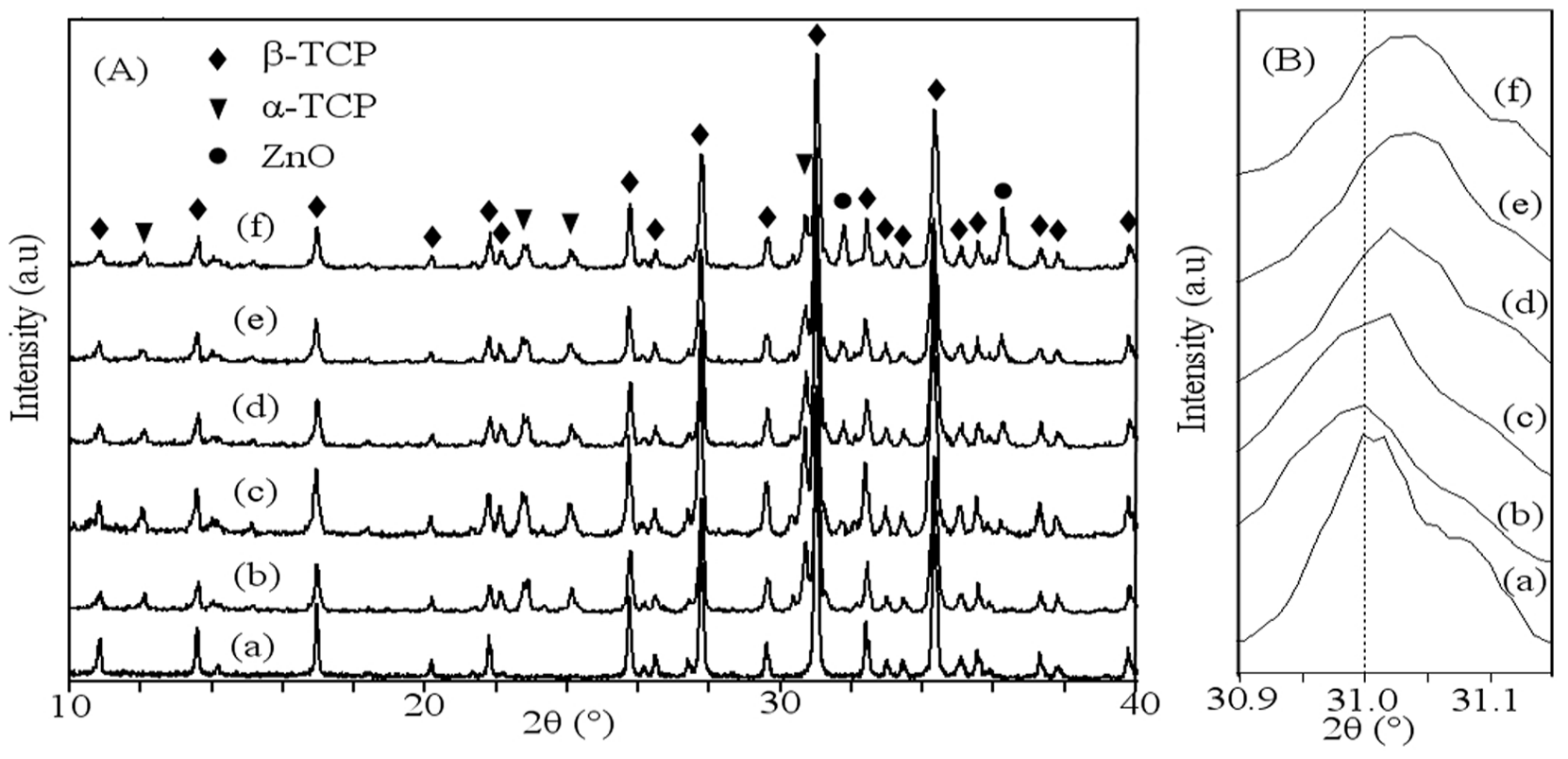
XRD patterns (A): 2θ from 10 to 40° and (B): 2θ from 30.9 to 31.15° of (a) the raw β-TCP powder, the scaffolds with different ratio of ZnO: (b) 0 wt%; (c) 0.5 wt%; (d) 1.5 wt%; (e) 2.5 wt% and (f) 3.5 wt%. (β-TCP, JCPDS file number 09-0169; α-TCP, JCPDS file number 09-0348; ZnO, JCPDS file number 36-1451).

The XRD patterns of raw β-TCP powder and the prepared scaffolds with different contents of ZnO were plotted in Fig. 5. Peaks of each XRD pattern were recorded and verified using standard JCPDS files. The obtained XRD pattern of raw β-TCP powder was shown in Fig. 5A (a). The XRD pattern of raw powder was the same as the pattern of pure β-TCP, and no impurity phase was observed according to JCPDS file number 09-0169. The patterns of the scaffolds with 0–3.5 wt% ZnO doped were shown in Figs. 5A (b–f). The results revealed that the intensity of ZnO peaks became stronger with the increase of ZnO content. A few small peaks of α-TCP were observed in the XRD patterns, indicating transformation of a small amount of β-TCP to α-TCP. JCODS file number 09-0348 and 36-1451 were used to identify the peaks of α-TCP and ZnO, respectively. The characteristic peaks of ZnO were visible in all ZnO doped scaffolds. A small shift in main diffraction peak position of β-TCP was observed for ZnO doped scaffolds (Fig. 5B). It means that the Zn^2+^ ions were substituted for the Ca^2+^ ion positions of the β-TCP structure, which was in good agreement with previous studies [29]–[30].

### Mechanical Properties

**Figure 6:**
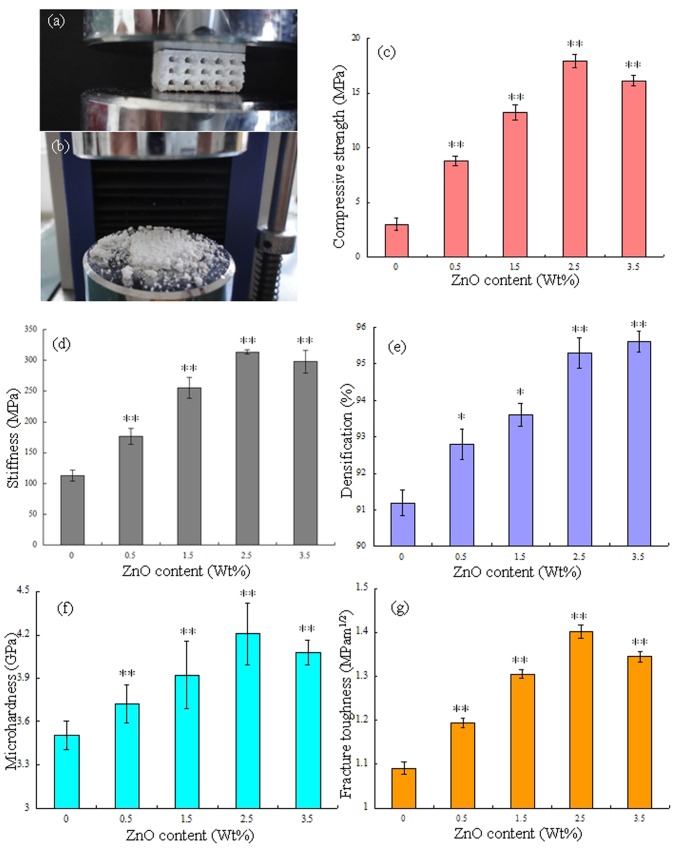
Representative porous TCP scaffold under compression test (a); overview of scaffold after compression test (b); Effect of ZnO contents on the compressive strength (c) and stiffness (d) of porous scaffold; Effect of ZnO contents on the densification (e), microhardness (f) and fracture toughness (g) of the strut. Data represents mean ± standard deviation for *n = *6, *P<0.05 and **P<0.01 (compared with pure TCP scaffold).

Compressive tests were carried out to evaluate the influence of ZnO content on the compressive strength and stiffness of the scaffold. The representative porous scaffold under compression test and after compression test was shown in Fig. 6 (a) and (b). The porous scaffolds were compressed between two circular platens that were sufficiently larger than the samples (Fig. 6a). The porous scaffolds were completely crushed after the compressive tests (Fig. 6b). The mechanical properties such as compressive strength, stiffness, densification, Vickers microhardness and fracture toughness were shown in Figs. 6c–g. With the content of ZnO increasing from 0 to 2.5 wt%, the compressive strength and stiffness of the scaffold increased from 3.01 to 17.89 MPa (Fig. 6c) and from 112.86 MPa to 313.48 MPa (Fig. 6d), respectively. The densification, microhardness and fracture toughness also increased from 91.2 to 95.3% (Fig. 6e), from 3.51 GPa to 4.20 GPa (Fig. 6f) and from 1.09 MPam^1/2^ to 1.40 MPam^1/2^ (Fig. 6g), respectively. The densification of the strut was 95.6% with ZnO content of 3.5 wt%. While the microhardness, fracture toughness, compressive strength and stiffness decreased to 4.08 GPa, 1.35 MPam^1/2^, 16.12 MPa and 297.74 MPa, respectively. The compressive strength was close to the highest strength reported for human cancellous bone [37] (Table 1). The highest value of stiffness was close to the higher limit of cancellous bone (0.05–0.5 GPa) [38] but far from that of cortical bone (7–30 GPa) [33]. The maximum micro hardness was about 6 times higher than that of human bone, and the maximum fracture toughness was a little lower than that of cortical bone [34]–[35]. The mechanical properties (compressive strength, stiffness, Vickers microhardness and fracture toughness) of all ZnO doped scaffolds were significantly higher than that of pure TCP scaffold (P<0.01). Viswanath et al. [40] used the micro and nano indentation techniques to investigate the mechanical response of flux-grown β-TCP single crystals. The values of fracture toughness were measured using the longest and smallest crack lengths vary from 0.41 to 1.15 MPam^1/2^, respectively. Zhang et al. [41] had applied the spark plasma sintering technique to fabricate macro porous β-TCP scaffolds from nano crystalline powders. The compressive strength of the sintered scaffolds with 66±1.0% and 55±1.0% porosity was 3.1±0.4 and 7.0±0.8 MPa, respectively. Park et al. [42] had performed the compression tests of the porous β-TCP scaffold specimens to obtain the stiffness. The stiffness of the porous scaffold with 50.9% porosity was 0.20–0.24 MPa.

**Table 1:** Summary of the properties of human bone.

	Density	Compressive strength	Stiffness	Fracture toughness	Microhardness
	(g/cm^3^)	(MPa)	(GPa)	(MPam^1/2^)	(GPa)
Cortical bone	1.7–2.0	130–180	7–30	2–12	0.62–0.74
Reference	[31]	[32]	[33]	[34]	[35]
Cancellous bone	0.03–0.12	0.1–16	0.05–0.5	–	0.63±0.11
Reference	[36]	[37]	[38]	–	[39]

These results revealed that ZnO could improve fracture toughness, compressive strength, stiffness and hardness of the scaffolds. The densification of the strut increased significantly with ZnO content increasing from 0 to 2.5 wt%, while it kept nearly the same when the ZnO content further increased to 3.5 wt%. The increase of the densification was most likely related to the presence of ZnO involved in the formation of the liquid phase that promoted high densification [21], [43]. It is widely accepted that the increase of sintered density is the most effective way to improve the mechanical properties of sintered parts. The increased fracture toughness, compressive strength, stiffness and hardness may be due to the increase of sintered density of the strut. The grain size decreased with ZnO content increasing from 0 to 2.5 wt%, while the grain size increased when ZnO content increases to 3.5 wt%. Meanwhile, the mechanical properties were associated with the average grain size, the larger the grain size, the lower the mechanical properties. The fracture toughness, compressive strength, stiffness and hardness decreased due to the sudden increase in grain size when the content of ZnO was 3.5 wt%. Therefore, the scaffold with 2.5 wt% ZnO doping was optimal due to the combined effects of densification and grain size.

### Cell Morphology on Scaffolds

**Figure 7:**
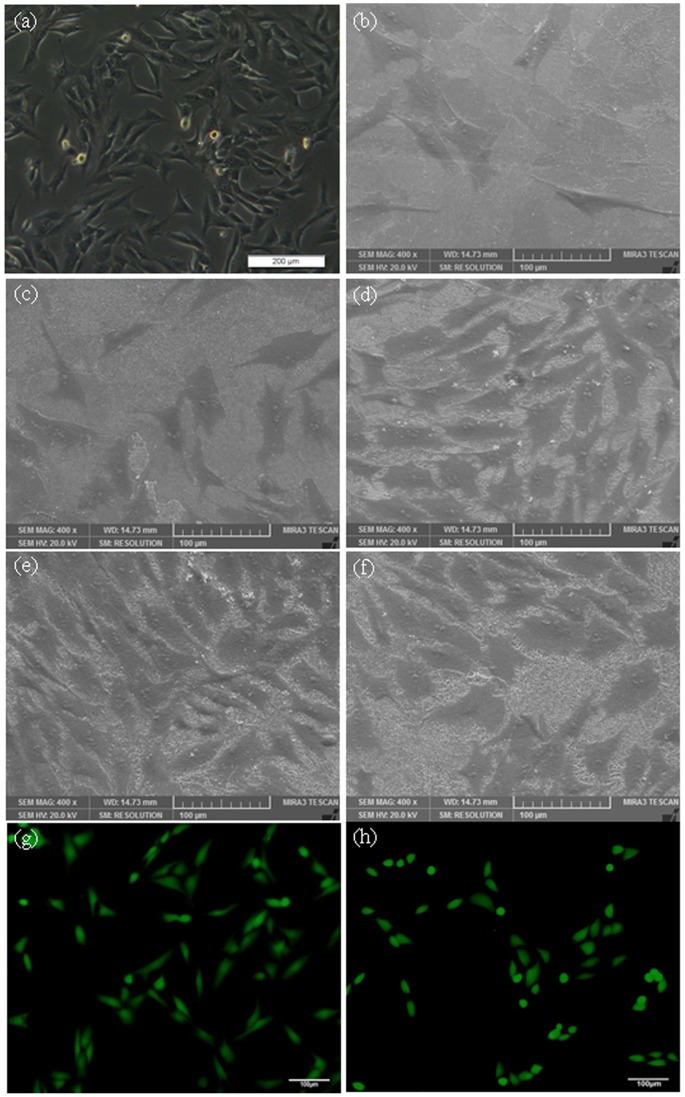
The morphology of initial MG-63 cells visualized by light microscopy (a), the morphology of MG-63 cells cultured on the scaffolds after 5 days by SEM (b) pure TCP; (c) 0.5 wt% ZnO; (d) 1.5 wt% ZnO; (e) 2.5 wt% ZnO and (f) 3.5 wt% ZnO, the fluorescence microscope images of MG-63 cells before (g) or after (h) incubation on the scaffolds with 2.5 wt% ZnO doping.

The morphology of initial MG-63 cells visualized by light microscopy was shown in Fig. 7 (a). The cell morphology showed the classical fusiform shape and the size of cell was about several tens of microns. The morphologies of MG-63 cells on the surface of the scaffolds after 5 days of culture were shown in Fig. 7 (b–f). It was obviously different in the response of the cells to the different scaffold. The cells attached and proliferated well on the surface of the scaffolds when the content of ZnO was 2.5 wt% (Fig. 7e). The cells were connected together and covered most of the surfaces. While the scaffold with 3.5 wt% ZnO showed lower cell density and fewer cells adhesion (Fig. 7f). The fluorescence microscope images of MG-63 cells before and after incubation on scaffolds with 2.5 wt% ZnO doping were shown in Fig. 7 (g) and (h). It was indicated that the MG-63 cells were viable, adhered and proliferated well on the scaffold. The results showed that ZnO had a stimulating effect on osteoblastic cell proliferation. However, the scaffolds showed reduced ability to support cell attachment and proliferation when ZnO content was over 2.5 wt%. It was reported that Zn promoted the activity of osteoblasts and/or inhibits the differentiation of osteoclasts, while it was harmful to cells when Zn concentration was too high [16], [44]. Bandyopadhyay [22] reported that higher ZnO doping had deleterious effects on cell attachments and growth, and cell apoptosis was observed for 3.5 wt% ZnO doped TCP scaffolds.

### Degradation and Bioactivity

**Figure 8:**
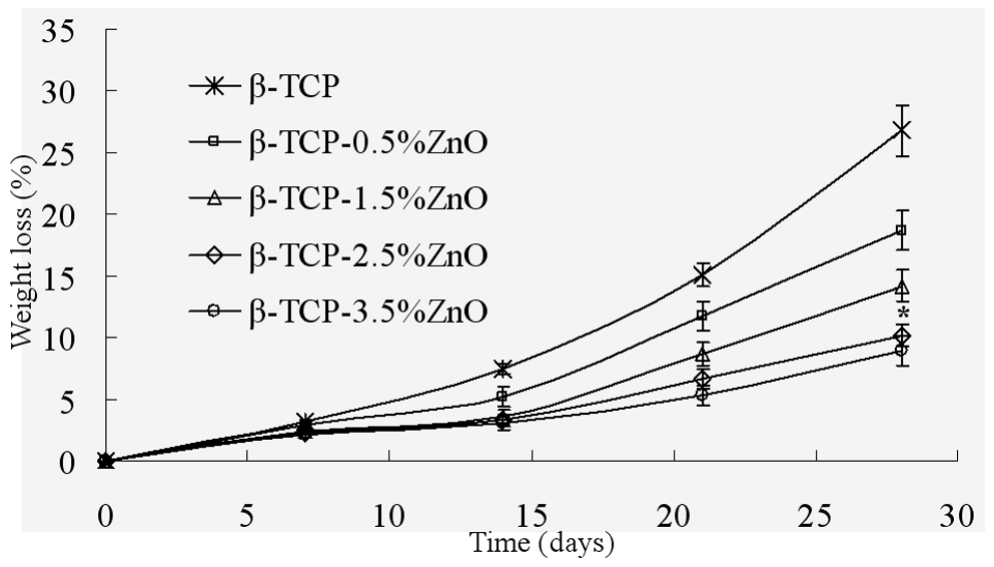
Weight loss assay of the scaffolds after incubation with SBF for different days. Data represents mean ± standard deviation for *n = *6, *P<0.05 (compared to pure TCP scaffold).

The degradation rate of the scaffolds with different ZnO content was measured in terms of their weight loss at different time points (day 7, 14, 21, 28) after immersion in SBF (Fig. 8). It was evident that there was an increase in weight loss with time for all scaffolds. The degradation rate of the scaffolds with 2.5 wt% ZnO doping was much lower than that of pure TCP scaffolds after soaking in SBF for 28 days (P<0.05). ZnO could inhibit weight loss rate of the β-TCP scaffolds and delay the degradation rate in SBF solution.

**Figure 9:**
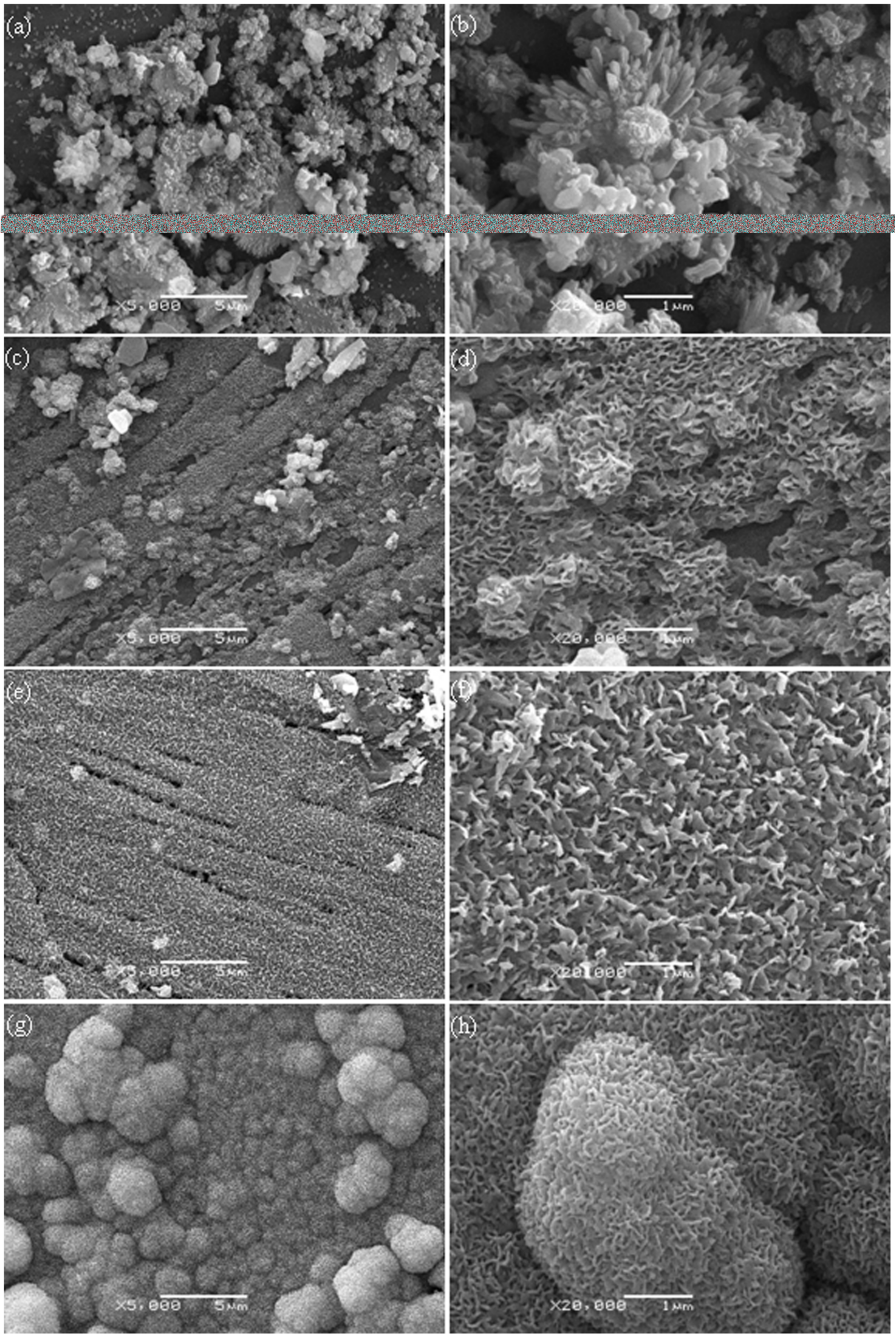
SEM images of bone like apatite layer formation on the surface of scaffolds with 2.5% ZnO doped after SBF incubation for 7 (a, b), 14 (c, d), 21 (e, f), 28 (g, h) days.

As the scaffold with 2.5 wt% ZnO doping possessed good mechanical properties and excellent biocompatibility, we chose the β-TCP scaffold with 2.5 wt% ZnO doping for the bioactivity assay. The scaffolds after soaked in SBF for different time interval was observed (Fig. 9). There was an increased apatite deposition with a more crystal structure on the surface of scaffolds with the time. Numerous tiny granular apatite particles were present on the surface of scaffolds and the particles were worm-like after soaking for 7 days (Figs. 9a, b). The worm-like apatite was more obvious and the crystal layers became more compact after soaking in SBF for 14 days (Figs. 9c, d). The surface was completely covered by a rough deposit layer, and the apatite layer was composed of numerous flake-like crystals on day 21 (Figs. 9e, f). Sponge-like crystals of apatite layer formed after immersion for up to 28 days (Figs. 9g, h). The formation of a bone-like apatite layer on biomaterials was assumed to be the precondition for their osteoinduction to induce bone formation. The ability to form apatite layers in SBF had been regarded as the evidence of bioactivity for bio ceramics [45]–[47].

**Figure 10:**
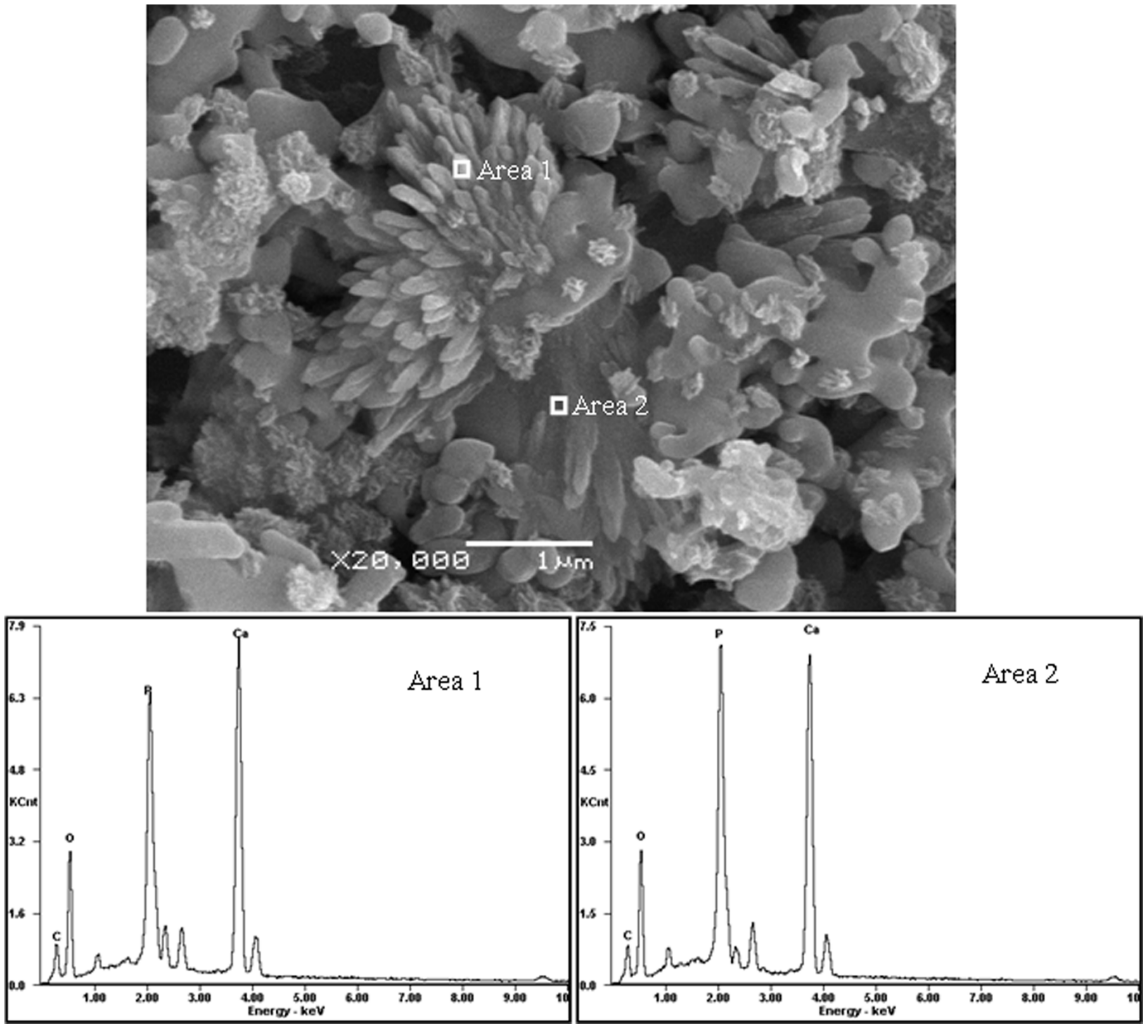
Images under SEM and EDX traces of the scaffold with 2.5% ZnO doped after SBF incubation for 7 days.

**Figure 11:**
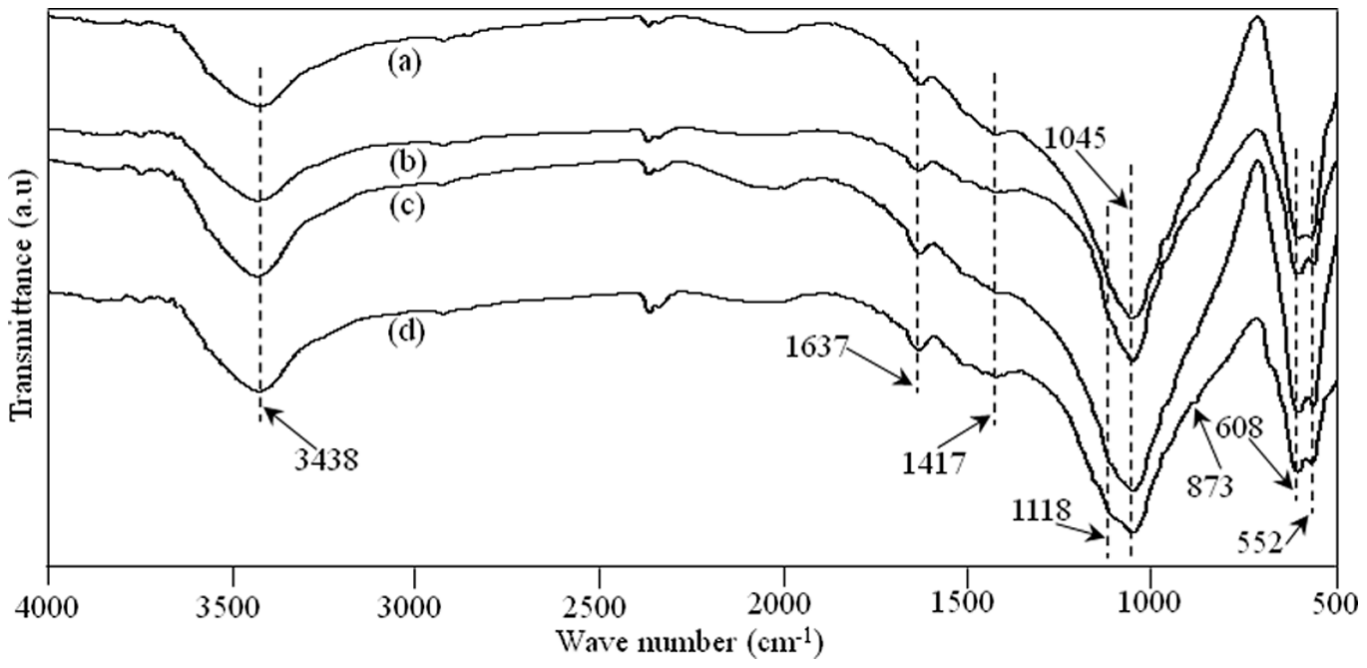
FT-IR spectra of the scaffold with 2.5 wt% ZnO doped after SBF incubation (a) 7 days; (b) 14 days; (c) 21 days and (d) 28 days.

The morphology and elemental composition of the apatite deposition on the scaffold with 2.5 wt% ZnO doping after 7 days in SBF were analyzed with SEM and EDX (Fig. 10). The strong peaks of oxygen, phosphorus, and calcium plus a weaker peak of carbon were observed in the spectra. The existence of carbon in the apatite implied that the layer might be carbonate-containing apatite phase, which could be further confirmed through FT-IR analysis. The FT-IR absorption spectra of the scaffold with 2.5 wt% ZnO doping after immersion in SBF for 7, 14, 21 and 28 days were shown in Fig. 11. The characteristic peaks of phosphate for β-TCP at wave numbers of 1118, 1045, 608, and 552 cm^−1 ^were all present. The peaks located at 873 and 1417 cm^−1^ were the vibration mode of carbonate [26]. It was possible that the carbonate replaced phosphate ions during the crystal growth process. The scaffolds could develop a bone like apatite layer on the surface when soaked in SBF. Such bone like apatite layer was believed to be beneficial to increase cell adhesion, proliferation and bone formation.

## Conclusions

Interconnected porous β-TCP scaffolds improved by ZnO were successfully prepared with SLS technique. The scaffolds were then tested for microstructure, mechanical, and biological properties. It was found that the addition of ZnO improved the densification of the strut of β-TCP scaffolds, and the average grain size of the scaffold decreased when ZnO content increased from 0 to 2.5 wt%, while the average grain size increased with higher content of ZnO. The minimum average grain size was 0.29 µm when the content of ZnO was 2.5 wt%. The addition of ZnO could improve the mechanical properties of fracture toughness, compressive strength, stiffness and hardness. The increase in mechanical properties could be due to the combined effects of densification and grain size. In vitro cell tests showed that the scaffold with 2.5 wt% ZnO doping exhibited a good cellular viability as well as cellular proliferation. The biodegradability tests revealed that the degradation rate of the scaffolds slowed down with the increase of ZnO content. The formation of a new bone like apatite layer indicated that the scaffold demonstrated better bioactivity. So β-TCP porous scaffolds with 2.5 wt% of ZnO using SLS technique may be a good candidate for the application of bone tissue engineering.
